# Radiological Diagnosis of a Rare Prepontine Lesion: Ecchordosis Physaliphora

**DOI:** 10.7759/cureus.24335

**Published:** 2022-04-21

**Authors:** Nilanjan Sarkar, Somen Chakravarthy, Rohit Chakravarty, Sandipan Mukhopadhyay

**Affiliations:** 1 Radiology, Tata Main Hospital, Jamshedpur, IND

**Keywords:** mri, chordoma, hamartoma, prepontine, ecchordosis physaliphora

## Abstract

Ecchordosis physaliphora (EP) is a notochordal remnant tissue rarely encountered during routine clinical practice. These lesions usually do not produce any significant symptoms as they are slow-growing and mostly small in size. Symptoms are due to mass effects on adjacent structures when they are large or extra-tumoral hemorrhage. Because of histological similarity with chordoma, diagnosis is challenging, and this differentiation is essential as the disease course and treatment differ significantly. Imaging plays a crucial role in identifying and distinguishing these lesions.

We report the case of a 16-year-old male who presented with intermittent headache and neck pain for six months. His routine clinical examinations were within normal limits. On neurological assessment, there was no focal neurodeficit. Evaluation of cranial nerves did not reveal any evidence of palsy. Routine hematological tests were also normal. A computed tomography (CT) scan of the brain revealed a mass in front of the pons. Magnetic resonance imaging (MRI) for further evaluation revealed a T1 hypointense and T2/fluid-attenuated inversion recovery hyperintense lesion in the pre-pontine cistern. There was no enhancement in the mass either in the post-contrast CT or MRI scans. There was no bony erosion and clivus was normal. Based on the location and characteristic imaging features, a diagnosis of EP was made.

There may be several other lesions that may present as a mass in the pre-pontine region. Histopathological tests may find it difficult to distinguish between lesions that originate from notochord remnants. Imaging studies play a vital role in confirming the diagnosis and help in planning treatment and follow-up.

## Introduction

Ecchordosis physaliphora (EP) is an extremely rare hamartomatous lesion characteristically located in the pre-pontine cistern of the brain. The term “hamartoma” is derived from two Greek words, hamartia, which means “defect or fault,” and -oma, which means a tumor. It is defined as a developmental error or malformation made of an unnatural mixture of cells and tissues which may be seen in many areas of the body. Hamartomatous lesions in routine clinical practice often lead to a diagnostic dilemma. These are usually found incidentally during imaging studies advised for other indications. These lesions do not require any treatment or intervention but distinguishing them from neoplastic lesions produces a diagnostic challenge. Misdiagnosis is possible that may lead to unnecessary interventions, adding to morbidity and mortality. These lesions may look like a tumor but usually do not undergo malignant transformation. Radiological investigations are used as a first line for their evaluation and are usually confirmatory. Histopathological examinations are usually reserved for cases in which radiological features are ambiguous. EP is a benign entity but histologically similar to malignant lesions such as clivus chordoma. Here, we present a case of EP in a 16-year-old boy who was investigated for headache. The diagnosis was made on characteristic imaging features on computed tomography (CT) and magnetic resonance imaging (MRI) scans.

## Case presentation

A 16-year-old boy presented with intermittent headache and neck pain for six months. The headache was a mild, dull ache with a sensation of tightness around the forehead. It was not associated with nausea, vomiting, vertigo, photophobia, or blurred vision. Pulse was regular with a normal rate. Blood pressure in the sitting position was within normal limits. There was no papilledema on fundoscopic evaluation. There was no evidence of raised intracranial tension or focal neurodeficit. Routine hematological examinations were normal. A provisional diagnosis of stress headache was made. However, before starting any treatment, a CT scan of the brain was advised.

**Figure 1:**
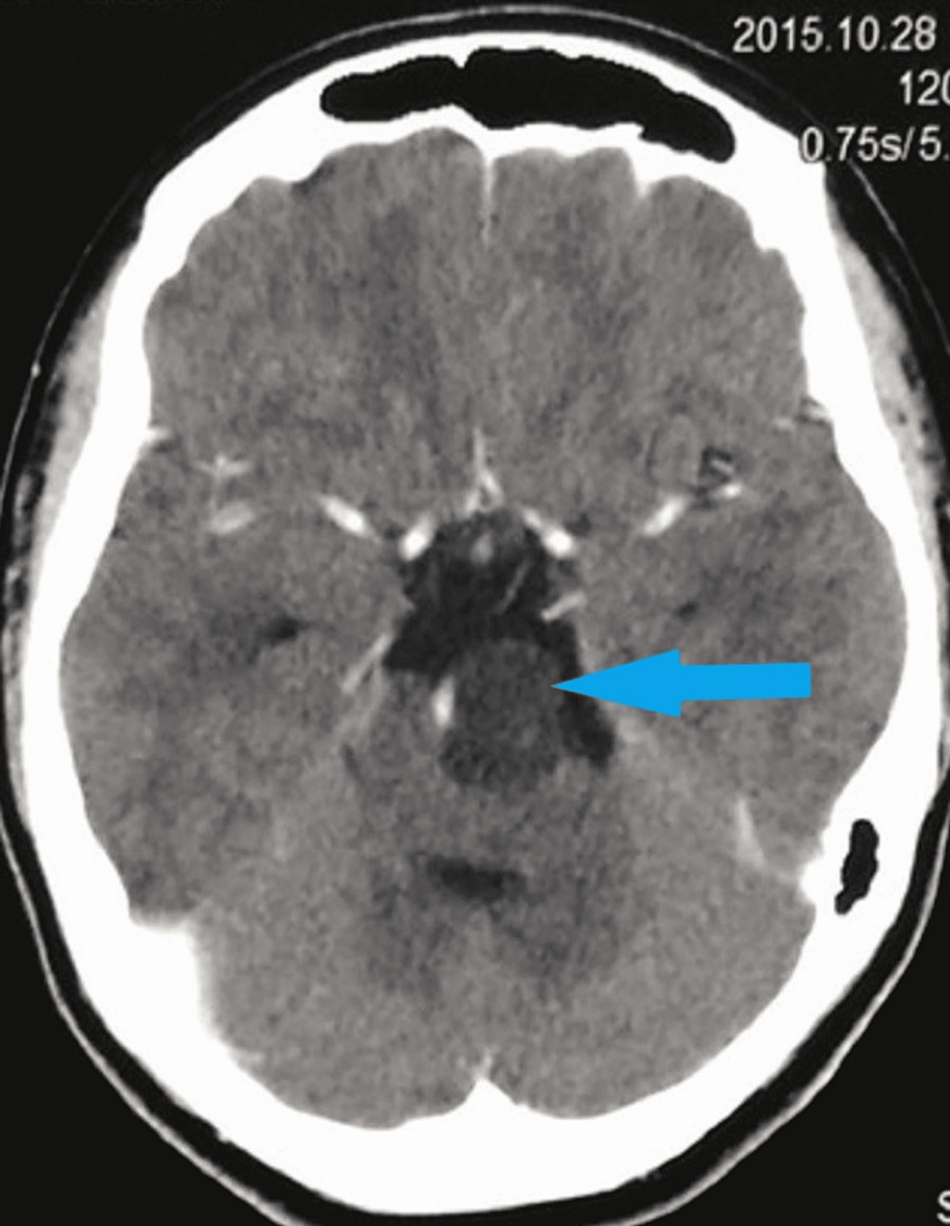
Contrast CT scan of the brain shows a non-enhancing hypodense lesion in the pre-pontine cistern. CT: computed tomography

Pre and post-contrast CT scans of the brain revealed a hypodense mass in front of the pons and compressing it. No enhancement was seen after administration of the intravenous contrast agent. Clivus appeared normal. The rest of the brain structures were also normal (Figure 1). The absence of contrast enhancement and lack of bony erosion or sclerosis ruled out the possibility of chordoma. Hence, three differential diagnoses, namely, arachnoid cyst, dermoid, and epidermoid were considered. Arachnoid cyst was excluded as the density of the lesion was not identical to cerebrospinal fluid (CSF). Dermoid contains fat and calcification and epidermoids are common in cerebellopontine angle locations; however, their possibility could not be excluded completely. Because no specific diagnosis could be obtained using the CT scan, further evaluation by MRI of the brain was suggested.

**Figure 2:**
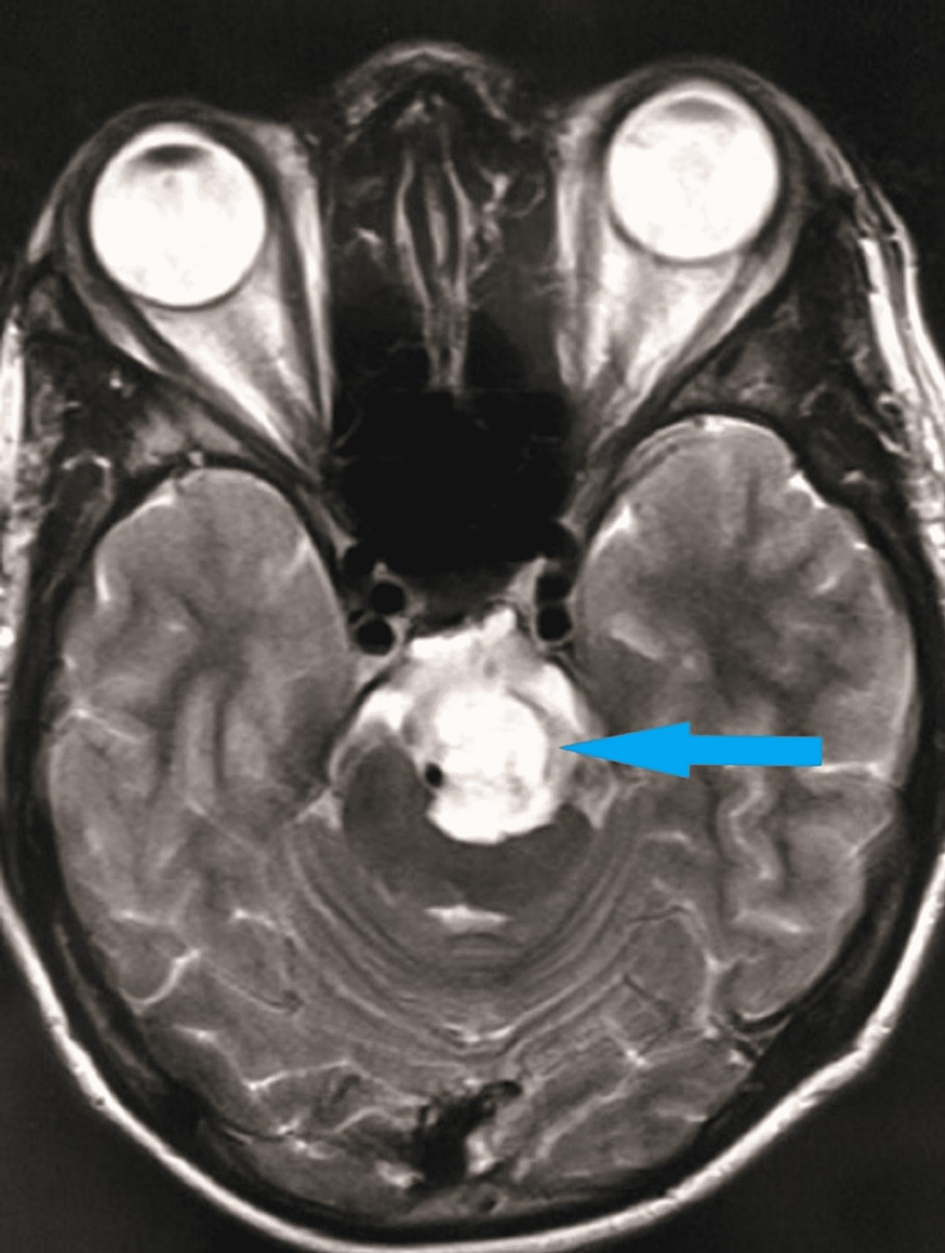
T2 axial MRI scan of the brain shows a hyperintense lesion in the pre-pontine cistern indenting the brainstem. MRI: magnetic resonance imaging

**Figure 3:**
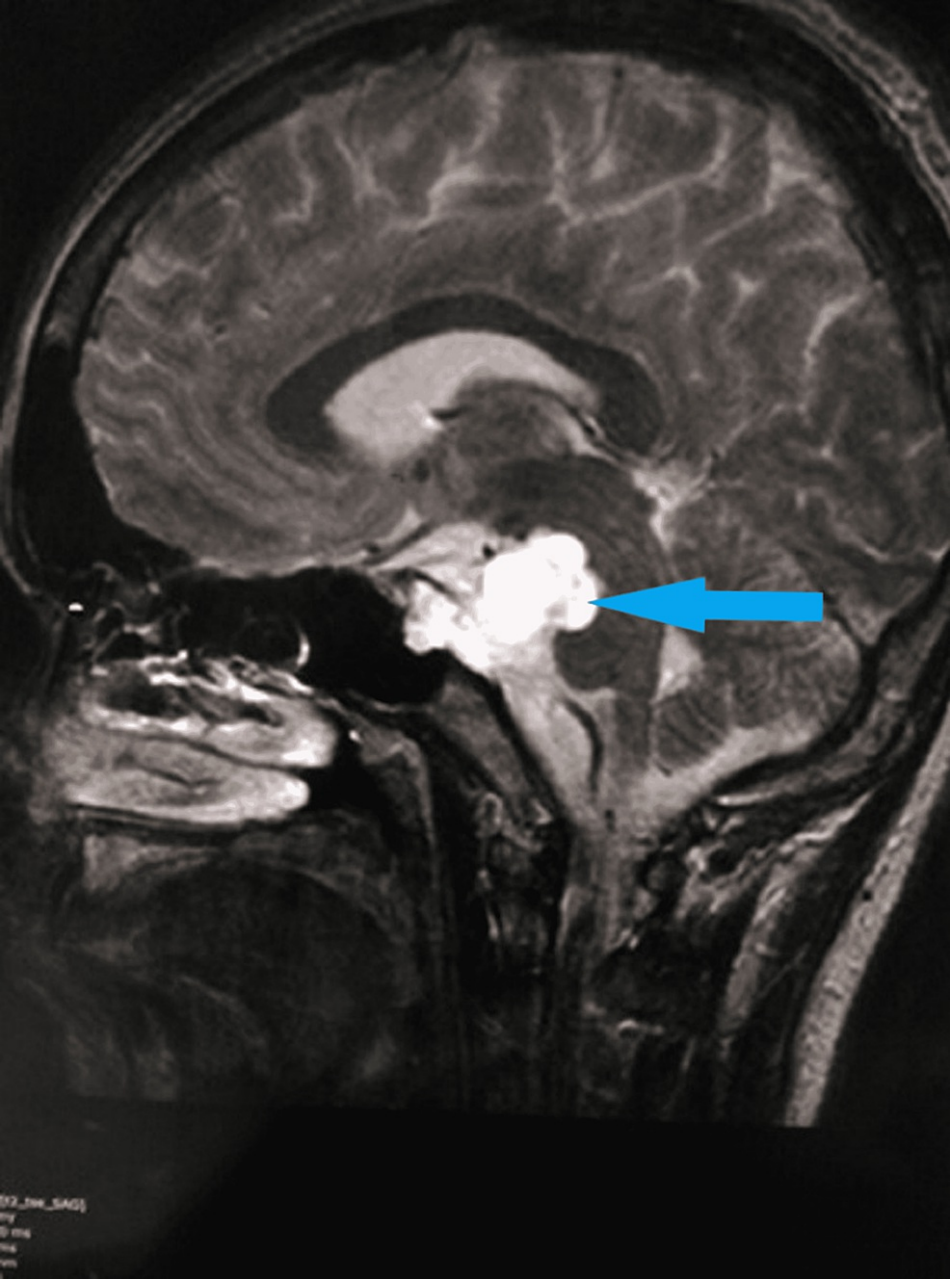
T2 sagittal MRI scan of the brain shows a hyperintense lesion in the retroclival region without any bony change. MRI: magnetic resonance imaging

**Figure 4:**
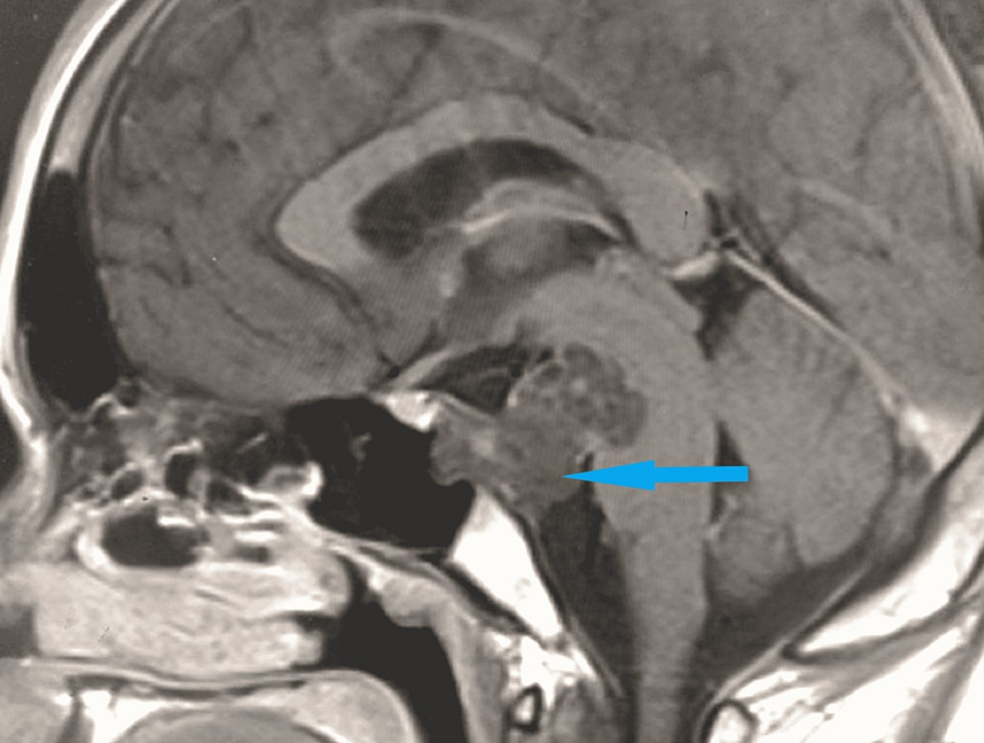
T1 sagittal post-contrast MRI scan of the brain shows no enhancement within the lesion. MRI: magnetic resonance imaging

MRI scan of the brain revealed a T2 hyperintense lesion in front of the pons in the pre-pontine cistern in the axial (Figure 2) and sagittal (Figure 3) sections. The lesion measured 1.6 cm × 1.4 cm × 1.3 cm. There was no restriction in diffusion which excluded the possibility of epidermoid. On the post-contrast T1 scan, there was no enhancement within the lesion (Figure 4). Based on these imaging findings, a diagnosis of EP was made as the lesion is typically located in the midline, in the pre-pontine cistern, without any appreciable enhancement in the CT scan or MRI and without any bony change. Because the imaging features were characteristic, surgical intervention was not considered. Imaging features in CT and MRI scans effectively ruled out other differentials.

The patient was advised symptomatic treatment. He was followed up, and there were no new symptoms or changes in the appearance of the mass even after one year.

## Discussion

EP is a hamartoma that arises from notochord remnants. The notochord is the primitive skeleton of vertebrates. It regresses during fetal life. In the dorsum sellae and the sacrococcygeal region, the two ends of the skeleton, there may be remnants of the primitive notochord later in adult life.

Both EP and intradural chordoma may be seen in the pre-pontine location as they both arise from the notochord remnant. It becomes very difficult to distinguish them even through histopathology. CT has a distinct advantage in visualizing the osseous stalk in the dorsal wall of the clivus adjacent to the lesion. It may not be very well appreciated in MRI scans. This unique imaging feature is not seen in other retroclival lesions [1]. Lack of contrast enhancement on MRI and CT scan helps to differentiate EP from an intradural chordoma [2,3]. It is these characteristic features in imaging studies that help in clinching the diagnosis of hamartomatous lesions such as EP. Because of its higher resolution, MRI helps in precise localization and characterization of the lesion and is the investigation of choice. Mass effects on adjoining structures are better evaluated with MRI which not only shows anatomical compression but also the presence of edema. Follow-up of these cases is also done through MRI which can accurately assess any change in the size of the lesion as well as complications such as mass effect and hemorrhage. MRI also does not have any radiation-related risks similar to the CT scan. In the absence of no new imaging features, a contrast scan may not be necessary during follow-up.

EP in most cases does not produce any symptoms and is incidentally diagnosed during imaging studies as the lesions grow very slowly and are very small in size. Some symptomatic cases of retroclival EP have been reported in the literature such as fatal pontine hemorrhage and CSF fistula. Although some cases of EP have produced symptoms such as headache or dizziness, they are not caused by the lesion itself [4,5]. Most of these non-specific complaints are relieved after symptomatic treatment and reassurance.

There are several other but comparatively rare imaging differentials for skull base masses such as metastasis from distant sites, multiple myeloma, and lymphoma originating from the clivus itself. Meningioma and diffuse leptomeningeal glioneuronal tumor may arise from the pachymeninges and present as a mass lesion in this region. Nasopharyngeal carcinoma may invade and involve the skull base. Chondrosarcoma may also arise from petroclival synchondrosis and present as a pre-pontine mass lesion. Benign lesions such as fibrous dysplasia, ossifying fibroma, and Paget’s disease may also involve this region [6-9]. These lesions have a typical imaging appearance in imaging studies. They are usually enhanced in post-contrast CT or MRI scans. They also cause bony changes such as erosions or remodeling. The site of origin also becomes evident like in the case of nasopharyngeal carcinoma. The benign lesions do not have any associated soft tissue component. None of the lesions have associated osseous stalk.

Hence, the diagnosis of a pre-pontine mass by imaging is critical in routine medical practice for treatment planning. While management of chordoma involves extensive surgery and postoperative radiotherapy, EP does not require any surgical intervention. Surgery is only required if it is large and causes symptoms such as cranial nerve palsy or life-threatening hemorrhage.

## Conclusions

EP is a condition infrequently encountered in routine clinical practice and usually asymptomatic. However, symptomatic cases create a diagnostic dilemma and requires differentiation from its malignant counterpart, chordoma. Because the treatment of these conditions are significantly different and histopathology is of limited help, an accurate radiological diagnosis is necessary. Lack of contrast enhancement on CT and MRI scans and absence of extensive bony destruction help clinch the diagnosis.
